# *In vitro* and *in vivo* embryo production efficiency in Flemish and Holstein donor females

**DOI:** 10.1590/1984-3143-AR2023-0080

**Published:** 2023-10-30

**Authors:** Fabiano Carminatti Zago, Luís Fernando Schütz, Renato Pereira da Costa Gerger, Luís Henrique de Aguiar, César Augusto Pinzón-Osorio, Alceu Mezzallira, José Luiz Rodrigues, Fabiana Forell, Marcelo Bertolini

**Affiliations:** 1 Empresa de Pesquisa Agropecuária e Extensão Rural de Santa Catarina, Lages, SC, Brasil; 2 Centro de Ciências Agroveterinárias, Universidade do Estado de Santa Catarina, Lages, SC, Brasil; 3 Faculdade de Veterinária, Universidade Federal do Rio Grande do Sul, Porto Alegre, RS, Brasil

**Keywords:** breed conservation, *in vitro* fertilization, multiple ovulation, ovum pick-up, cattle

## Abstract

The aim of this study was to compare embryo production efficiency in Flemish and Holstein donor females using ovum pick-up and *in vitro* fertilization (OPU-IVF) or *in vivo* production (superovulation; SOV) procedures. The study was conducted using a split-plot design, with eight Flemish and eight Holstein non-lactating cycling females. Females were subjected to ten weekly OPU/IVF sessions and/or two SOV/embryo collections sessions at a 63-day interval, for a total of 160 OPU-IVF and 32 SOV sessions. Mean numbers of follicles and corpora lutea, and *cumulus*-oocyte complex (COC) recovery rates were similar between breeds after the OPU and SOV sessions. However, Flemish donors yielded better quality grade II COCs (301, 41.9%) than Holstein females (609, and 202, 33.1%). Also, cleavage and blastocyst rates, and the total number and the mean number of viable embryos obtained after OPU-IVF were higher in Flemish (49.6% and 11.8%, and 63 and 11.8 per donor, respectively) than in Holstein (32.8% and 7.2%, and 34 and 7.2 per donor, respectively) females. Flemish females were also more efficient in yielding viable embryos after SOV (111, 7.3 per donor) than Holstein (48, 3.3 per donor) females. Overall, Flemish donor females had better responses to OPU-IVF or SOV procedures than Holstein counterparts. Irrespective of the breeds, SOV procedures were more efficient than OPU-IVF in yielding more viable embryos, under the conditions of this study. Both reproductive procedures were useful tools for the genetic conservation of the Flemish cattle breed in Southern Brazil.

## Introduction

Cattle industry is a key component in global agriculture (Gicquel et al., 2020), with the value chain having an essential part in food supply and in supporting food security (Godde et al., 2021). Over the decades, great changes have occurred in the livestock industry and animal husbandry, leading to a progressive replacement of native breeds with high-yielding commercial cattle breeds to maximize the overall production and economic profit (Zander et al., 2013). However, such phenomenon was accompanied by significant decline in the diversity of native, ancient or even indigenous cattle breeds (Clasen et al., 2021).

In recent times, an increase in interest has been emerging for the preservation of breeds deemed to extinction (Liu et al., 2021), as superior production and functional traits have been recognized in some ancient or native populations when compared with commercial cattle (Parra et al., 2020).

Flemish cattle (*Bos taurus taurus*), also known as Rouge Flamande, is one of the oldest French breeds (Lauvie et al., 2008) characterized by its high fertility, maternal ability, sexual precocity, longevity and hardiness, with the production of milk with high fat and protein contents, and good quality meat (Thaler et al., 1996; Goetten et al., 2015). Due to the small size of the breed´s population, Flemish cattle have been targeted for conservation programs in France since late 1970s (Lauvie et al., 2008), as it is considered in risk of extinction (Marian et al., 2023). In Brazil, the breed was introduced in 1912, and currently, the remaining animals from the original group comprise a pure herd of approximately 50 animals, which has not been crossed with other breeds (Alfonzo et al., 2021). In this context, this group of animals represents a unique genetic resource for preservation and for the study of reproductive traits under the subtropical environment (Goetten et al., 2015).

For being a breed in risk (Taberlet et al., 2008), the use of OPU/IVF and SOV procedures in Flemish cattle can assist in preserving such genetic pool for the benefit of a future upturn. However, little is reported about the reproductive biology in Flemish cattle, with no reports available on the efficiency in terms of embryo production, either *in vivo* or *in vitro*, in such breed. Thus, the aim of this study was to evaluate the efficiencies of *in vivo* and *in vitro* embryo production systems, by OPU-IVF and SOV procedures, respectively, under the subtropical environment, comparing results in Flemish donors with Holstein females, a breed with a significant database in the literature.

## Methods

### Experimental design

A total of 16 multiparous non-lactating Flemish (n=8) and Holstein (n=8) cycling bovine females were assigned to two groups (A and B), in a split plot design, with four Flemish and four Holstein females in each, homogeneously distributed in terms of age, body condition score, weight, and number of lactations. Group A was subjected to 10 consecutive weekly OPU sessions followed by *in vitro* production (IVP) of embryos by IVF procedures (OPU-IVF). Simultaneously, females in Group B were subjected to two hormonal protocols, 63 days apart, for the *in vivo* production (IVD) of embryos by superovulation and artificial insemination (SOV) followed by embryo flushing on Day 7 of embryo development (AI = Day 0). Then, after a five-day resting period, treatments were switched between groups and at the end of the experiment, each animal of each breed underwent a total of ten OPU-IVF sessions and two SOV procedures, totaling 80 OPU-IVF and 16 SOV sessions per breed.

### Chemicals and reagents

Reagents were from Sigma-Aldrich Co. (St. Louis, MO, USA), unless stated otherwise.

### Animals and husbandry

Non-lactating cycling Flemish (n=8, 8.1 ± 4.3 years old) and Holstein (n=8, 6.2 ± 2.1 years old) females, weighing 583.0 ± 117.5 kg and 588.5 ± 79.1 kg, with body condition scores (BCS) of 2.4 ± 0.5 and 2.3 ± 0.6 (1 to 5), and after 3.3 ± 1.8 and 2.4 ± 0.7 lactations (2 to 5), respectively, were selected and assigned to each experimental group. Animals were held and maintained at the Santa Catarina State Agriculture Research and Extension Center, Lages, SC, Brazil (27° 48' 26.8” S and 50° 19' 55.1” W), where experiments (OPU and SOV) were carried out. Experiments followed the Brazilian regulations for animal ethics and use in research and were approved by the Ethics Committee on Animal Experimentation of the Center for Agroveterinary Sciences of the Santa Catarina State University (CAV/UDESC; protocol number 1.15.10./2010).

### *In vivo* recovery of *cumulus*-oocyte complexes (COCs) by OPU procedures and *in vitro* embryo production by IVF

Ovum pick up (OPU) was performed as previously described (Pieterse et al., 1988), and adapted from Seneda et al. (2001), with modifications. An ultrasound device (Falcon 100, Pie Medical, The Netherlands) coupled to a 6.0-MHz linear array transducer adapted to a transvaginal follicular aspiration guide (WTA, Brazil) was used to aspirate all &gt;4 mm follicles. Number and size (small, &lt;8,0 mm; medium, 8.0-11.0 mm; and large, &gt;11.0 mm follicles) of visible and effectively aspirated follicles were recorded per OPU session per female.

*In vitro* production (IVP) of embryos was performed according to Oliveira et al. (2006), with modifications. Briefly, recovered COCs were selected under a stereomicroscope (×15 magnification). Selected COCs, graded as I, II, III, and IV based on Stojkovic et al. (2001), were used for the experiments. Bovine COCs were also recovered from bovine ovaries collected at a regional slaughterhouse in parallel to the OPU sessions to be used as controls in the IVP procedures, according to Ribeiro et al. (2009). After selection, COCs were washed and transferred in groups of 10 to 15 to 50-μL drops of *in vitro* maturation (IVM) medium under mineral oil and were incubated for 22 to 24 h at 39°C, 5% CO_2_ in air and 95% relative humidity. The IVM medium consisted of TCM-199 (Earle's salts, Invitrogen^®^, Walthan, Massachusetts, USA), supplemented with 26.2 mM NaHCO_3_, 0.2 mM sodium pyruvate, 0,5 μg/mL FSH (Folltropin^®^, Bioniche, Canada), 0.5 μg/mL LH (Lutropin^®^, Bioniche, Canada) and 10% inactivated estrus mare serum (EMS).

Frozen-thawed bovine motile sperm cells were segregated by the Percoll method (Machado et al., 2009) and were added to droplets containing up to 10-15 COCs at a final concentration of 2 × 10^6^ sperm cells/mL in 50-μL drops of IVF medium, composed of TALP-Fert medium supplemented with 30 μg/mL heparin, 0.72 μg/mL penicillinamine, 0.26 μg/mL hypotaurine and 0.04 μg/mL epinephrine (Parrish et al., 1988). For IVF, sperm cells and COCs were co-incubated for 18 to 22 h at 39°C under 5% CO_2_ in saturated humidity (Day 0).

After IVF (Day 1), presumptive zygotes were gentle denuded by repeated pipetting in SOFaaci medium (Holm et al., 1999), then transferred into four-well dishes (Nunc, Roskilde, Denmark) in groups of 10-15 structures, in 50-μL of SOFaaci medium supplemented with 6 mg/mL BSA covered by mineral oil, to be *in vitro*-cultured (IVC) at 39°C and 5% CO_2_ in humidified air for up to Day 2 of development, when cleavage rates were evaluated. Then, plates were placed in the Foiled Bag system (Vajta et al., 1997) under a gas mixture containing 90% of N_2_, 5% of O_2_ and 5% of CO_2_ and saturated humidity, for IVC at 38.5°C up to Day 7 of development, when blastocyst rates were evaluated. In addition, embryo stage and morphological quality (grades I, II or III) were also assessed on Day 7, according to Ribeiro et al. (2009), as adapted from Stringfellow and Givens (2009).

### *In vivo* embryo production by SOV procedures

*In vivo*-derived (IVD) embryos were obtained after superovulation and AI of donor females, according to Bó et al. (2006). In brief, the superovulatory FSH treatment initiated on Day 4 after the insertion of an intravaginal progesterone (P4) device (1.55 g progesterone; PRID^®^, Ceva Sante Animale, France) and 2 mg estradiol benzoate (Estrogin^®^, Farmavet, Brazil) injection via IM (Day 0). Doses of FSH (Folltropin^®^) were given IM twice a day (am/pm) in eight decreasing doses over a 4-day period. The total FSH dose was based on age and body weight, with eight older females (&gt;7 years old) weighing &gt;550 kg; four 5- to 7-years old females weighing between 520 to 580 kg; and four younger females (&lt;5 years old) weighing between 480 a 520 kg receiving a total FSH dose of 350 mg, 300 mg and 250 mg, respectively, being evenly distributed between groups. A dose of 0.150 mg d-cloprostenol (Prolise^®^, Tecnopec Ltda., São Paulo, Brazil) was given IM along with the sixth (Day 6) and seventh (Day 7) FSH doses. The intravaginal P4 device was removed on Day 7. Estrous behavior was observed every 4 to 6 h starting on Day 8 of the protocol, and AI was performed at 12 and 24 h after the onset of estrus.

Embryos were non-surgically recovered on Day 7 of development (AI = Day 0) using D-PBS medium supplemented with 0.5% EMS, according to Cruz et al. (2008). Embryos were evaluated under a stereomicroscope and classified according to the developmental stage and morphological quality (Stringfellow and Givens, 2009).

Frozen-thawed semen from the same Flemish bull, previously proven fertile for AI, was used for embryo production by SOV and OPU-IVF procedures in both breeds, as above.

### Data analysis

Quantitative data were analyzed in a 2 × 2 factorial design, considering breed (Flemish, Holstein) and embryo production system (IVD, IVP) as main effects, with pairwise comparisons by the Tukey test (SAS^®^, SAS Institute Inc., EUA, 2002). The Shapiro-Wilk test was used for the analysis of data normality (Minitab^®^, State College, Pennsylvania, USA). Non-normal data were subjected to arc-sin [arc-sine (√(X/100))] or log [log (X+1)] transformations, when needed, and depending on the nature of the data. The Friedman test was used for non-parametric data (Proc Rank and Proc Mixed of SAS). Qualitative data regarding cleavage and blastocyst rates were compared between groups within each experiment by the χ^2^ test. Embryo kinetics and embryo morphology were compared by the Kruskal-Wallis test of Minitab. The level of significance was 5% (P&lt;0.05).

## Results

### *In vivo* recovery of *cumulus*-oocyte complexes (COCs) by OPU and *in vitro* embryo production by IVF

The mean numbers of &gt;4-mm ovarian follicles by size between breeds are shown in Table1. No differences were observed (P&gt;0.05) in the number of follicles per ovary according to follicular diameter in females from both breeds, with donor females having more small size follicles (&lt;8.0 mm, 89%) than medium (8.0-11.0 mm, 7%) and large (&gt;11.0 mm, 3%) size follicles. No differences were observed in the number of follicles and proportion of follicles by size according to follicular diameter between ovaries, irrespective of the breed.

**Table 1:** Mean number (LSM ± SEM) of visualized &gt;4-mm ovarian follicles prior to OPU procedures in eight Flemish and eight Holstein multiparous females after 10 consecutive weekly OPU sessions†.

**Visualized follicles/ovary**	**Flemish donors**	**Holstein donors**
**Small (&lt;8.0 mm)**	13.8 ± 0.7^aA^	13.8 ± 0.7^aA^
**Medium (8.0-11.0 mm)**	1.1 ± 0.2^aB^	1.2 ± 0.2^aB^
**Large (&gt;11.0 mm)**	0.4 ± 0.1^aB^	0.6 ± 0.1^aB^
**Total**	15.3 ± 0.7^a^	15.6 ± 0.7^a^

a,b: numbers with distinct superscripts in each row differ, for P&lt;0.05; A,B: numbers with distinct superscripts in each column differ, for P&lt;0.05. ^†^Total of 80 OPU sessions in females from each breed, with eight females per breed.

After 80 OPU sessions per breed, a total of 718 and 609 total retrieved COCs and 635 and 536 viable COCs (Grades I, II, and III) were recovered from Flemish and Holstein donor females, respectively (Table 2).

**Table 2:** Efficiency of OPU procedures in eight Flemish and eight Holstein multiparous females after 10 consecutive weekly OPU sessions†. Mean number (LSM ± SEM), total number and recovery rate or proportion (%) of aspirated follicles, and retrieved, viable, and morphologically graded *cumulus*-oocyte complexes (COCs).

**OPU output**		**Flemish donors**			**Holstein donors**		
		**Mean**	**n**	**%**	**Mean**	**n**	**%**
**Aspirated follicles**		12.3 ± 0.4^a^	961^a^	-	11.4 ± 0.4^b^	860^b^	-
**Retrieved COCs**		9.2 ± 0.7^a^	718^a^	74.7^a^	8.2 ± 0.7^a^	609^b^	70.8^a^
**Viable COCs**‡		8.0 ± 0.7^a^	635^a^	66.1^a^	7.3 ± 0.7^a^	543^b^	63.1^a^
**COC by grade**	**I**	0.6 ± 0.2^a^	54^a^	7.5^aC^	0.6 ± 0.2^a^	48^a^	7.9^aC^
	**II**	3.6 ± 0.4^a^	301^a^	41.9^aA^	2.5 ± 0.4^b^	202^b^	33.1^bB^
	**III**	3.8 ± 0.5^a^	280^a^	39.0^aA^	4.1 ± 0.5^a^	293^a^	48.2^bA^
	**IV**	1.2 ± 0.2^a^	83^a^	13.1^aB^	0.9 ± 0.2^a^	66^a^	10.8^aC^

a,b: numbers with distinct superscripts in each row differ, for P&lt;0.05, according to each type of data in comparison (mean, total number, or recovery rate); A,B,C: numbers with distinct superscripts in each column differ, for P&lt;0.05. †Total of 80 OPU sessions in females from each breed, with eight females per breed. ‡Grades I, II and III.

**Figure 1:**
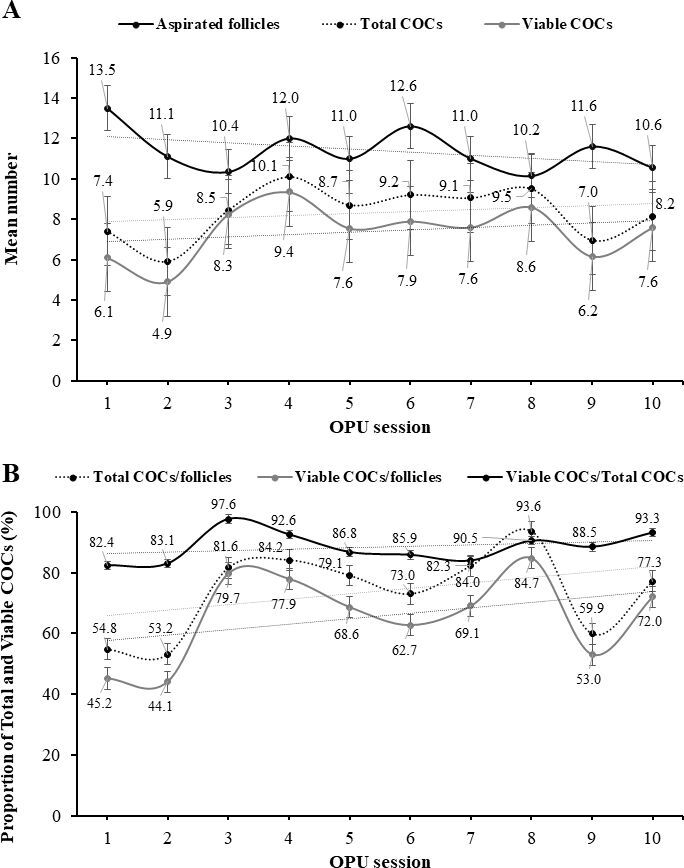
Mean number of aspirated follicles and mean number (A) and proportion (B) of total and viable retrieved *cumulus*-oocyte complexes (COCs) per weekly session of OPU procedure, as a mean for eight Flemish and eight Holstein multiparous females. Trendlines: dotted straight lines.

Overall, the total number and the mean number of aspirated follicles and the total number of recovered and viable COCs obtained by OPU were greater in females from the Flemish breed than in Holstein females. However, mean numbers and proportion of retrieved and viable COCs were similar between females from both breeds. On a per OPU session, mean number of aspirated follicles per female, and the mean number and proportion (recovery rate, %) of retrieved total COCs and viable COCs per female were similar between females from both breeds, ranging from 8.6 to 15.4 follicles, and 7.0 to 11.3 (44.2 to 94.9%) total COCs, and 3.0 to 9.9 (34.9 to 90.8% and 74.1 to 100%) viable COCs, respectively (Figure 1).

On average, 88.4% of the retrieved COCs were graded viable (Grades I, II, and III), irrespective of the breed and OPU session, with no differences between groups and OPU sessions, although better grade COCs were retrieved from Flemish donor females. A small proportion of grade I COCs were collected from females from both breeds than the other grades, with most COCs (close to 80%) falling within grades II and III in donors from both breeds. However, more grade II COCs (mean and total number and proportion) were collected from Flemish donors than Holstein females (Table 2). In turn, although not different in terms of mean and total numbers, Holstein females had a higher proportion of total grade III COCs than Flemish females, and than the other grades, within the same breed. Such differences may have affected embryo yield and development, as below.

A total of 1,122 COCs were used for IVF, with 608, 514, and 641 COCs from Flemish and Holstein donors, and from slaughterhouse ovaries, respectively. Cleavage and blastocyst rates were higher in the IVP control group (P&lt;0.05) than the other groups, which were different from one another (Table 3). When blastocyst rates were compared based on the number of cleaved embryos, no differences were observed in embryo development from females from both breeds, demonstrating that differences mostly relied on cleavage rates, being lower in embryos from Holstein females, with developmental rates following cleavage to the blastocyst stage being rather low, irrespective of the breed.

**Table 3:** *In vitro* embryo development to the blastocyst stage between eight Flemish and eight Holstein females following *in vitro* embryo production (IVP) by *in vitro* fertilization (IVF) procedures after 10 consecutive weekly OPU sessions†, as compared with IVP/IVF using slaughterhouse ovaries as controls (IVP control).

**Breed base**	**IVC**		**Cleavage rate**		**Blastocyst rate**	
	**n**	**n**	**%**	**n**	**%^ɛ^**	**%^¥^**
**Holstein donors**	467	153	32.8^c^	34	7.2^c^	23.4^b^
**Flemish donors**	532	264	49.6^b^	63	11.8^b^	24.0^b^
**IVP control**‡	596	364	61.1^a^	169	28.4^a^	46.4^a^

a,b,c: numbers with distinct superscripts in each column differ, for P&lt;0.05. †Total of 80 OPU sessions in females from each breed, with eight females per breed. ‡From bovine slaughterhouse ovaries. ^ɛ^Based on the total number of structures in *in vitro* culture (IVC); ^¥^Based on the total number of cleaved embryos.

**Figure 2:**
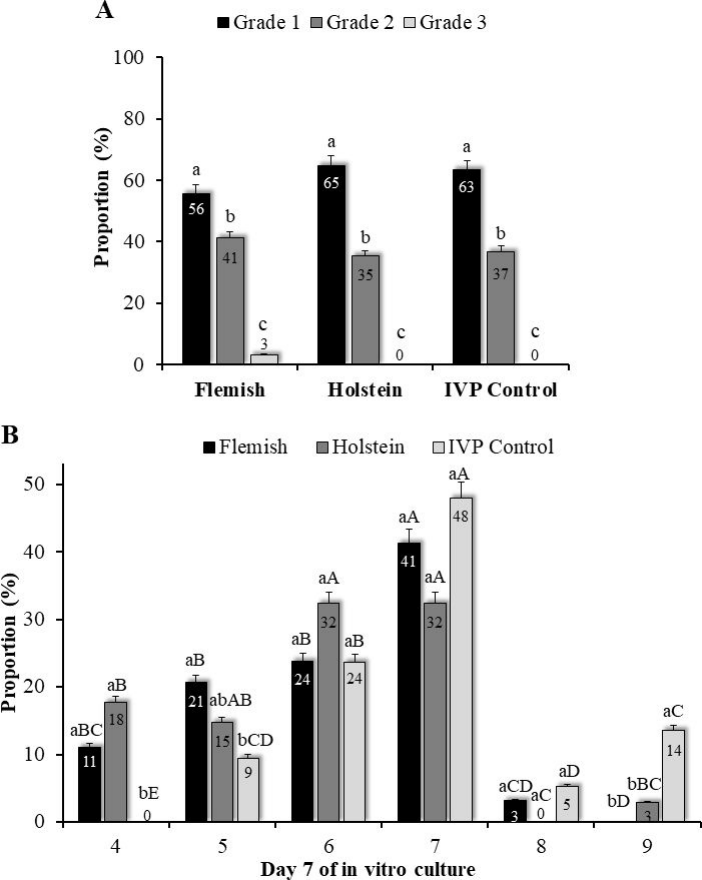
*In vitro* bovine embryo development (%) on Day 7 of *in vitro* culture (IVC) based on (A) morphological quality and (B) stage of development using COCs retrieved after 10 consecutive weekly OPU sessions from eight Flemish and eight Holstein females and from slaughterhouse ovaries (IVP controls). Morphological quality as grade 1: excellent; grade 2: good; grade 3: poor. Embryo stages as 4: compact morula; 5: early blastocyst; 6: blastocyst; 7: expanded blastocyst; 8: hatching blastocyst; 9: hatched blastocyst. a,b: columns with distinct superscripts within each stage of development between Flemish, Holstein and IVP Control embryos differ, for P&lt;0.05; A-E: columns with distinct superscripts between stages of development within each group (Flemish, Holstein, IVP Control) differ, for P&lt;0.05.

In all groups, the proportion of grade 1 Day-7 blastocysts was higher (P&lt;0.05) than grade 2 embryos, which in turn were higher than grade 3 blastocysts, with no differences between the IVP control and breed base (Figure 2A). Embryo kinetics on Day 7 of development was more advanced for IVP Control embryos, with more hatched blastocysts (9) and fewer compact morulas (4) and early blastocysts (5) than embryos from Flemish and Holstein females (except for stage 5 for Holstein embryos, which were similar to the other groups), with no differences in the breed base (Figure 2B). Nevertheless, proportion of embryos at the blastocyst (6), expanded blastocyst (7) and hatching blastocyst (8) stages were similar between groups. Most Day-7 embryos were at the blastocyst (6) and expanded blastocyst (7) stages.

### *In vivo* embryo production by SOV procedures

Tables 4, 5, and 6 summarize data regarding ovarian superstimulation response, embryo yield and embryo quality in Flemish and Holstein donor females following superovulation (SOV) procedures. Response to SOV, measured by the number of CLs estimated prior to uterine flushing, and the total number of recovered and viable structures were higher in Flemish donor females than in Holstein females (Table 4). Although the mean number of CLs per donor was similar between females from both breeds, lower (P&lt;0.05) mean numbers of recovered structures and viable embryos per donor were obtained in Holstein donor females than in Flemish donor females. In addition, efficiencies for the recovery of total structures and viable embryos, on a total of CL basis, were higher (P&lt;0.05) in Flemish donor females than in Holstein counterparts (Table 4). However, the proportion of viable embryos, based on the total number of recovered structures, and the mean number and proportion of degenerated embryos and unfertilized oocytes (UFO), as depicted in Table 5, were similar between females from both breeds. Overall, from a total of 32 SOV procedures for both breeds, a total of 377 CLs were detected, 258 structures were retrieved (68.4%) and 159 viable embryos were recovered (42.2% on a per total CL basis, and 61.6% on a total recovered structure basis), in a mean embryo yield of 5.0 embryos per donor, irrespective of the breed base.

**Table 4:** Efficiency of *in vivo* embryo production in eight Flemish and eight Holstein donor females following superovulation (SOV) procedures†.

**Donors**	**Total CLs** **‡**		**Recovered structures**			**Viable embryos**			
	**n**	**Mean**	**n**	**%**	**Mean**	**n**	**%** ^ɛ^	**%** ^¥^	**Mean**
**Holstein**	168^b^	11.3 ± 1.8^a^	80^b^	47.6^b^	5.4 ± 2.4^b^	48^b^	28.6^b^	60.0^a^	3.3 ± 2.0^b^
**Flemish**	209^a^	14.0 ± 1.8^a^	178^a^	85.2^a^	11.8 ± 2.4^a^	111^a^	53.1^a^	62.4^a^	7.3 ± 2.0^a^

a,b: numbers with distinct superscripts in each column differ (P&lt;0.05). ^†^Total of 16 SOV procedures in donors from each breed, with eight females per breed for two SOV procedures per female. ‡CLs: Corpora lutea, estimated by transrectal ultrasonography prior to embryo collection on Day 7 of development. ^ɛ^Based on the number of viable embryos by the number of CLs; ^¥^Based on the number of viable embryos by the number of recovered structures.

**Table 5:** Total number, mean number (LSM ± SEM) and distribution (%) of recovered structures obtained from eight Flemish and eight Holstein donor females following superovulation (SOV) procedures†.

**Donors**	**Viable**			**Degenerated**			**UFO** **‡**		
	**n**	**%**	**Mean**	**n**	**%**	**Mean**	**n**	**%**	**Mean**
**Holstein**	48	60.0^aA^	3.3 ± 2.9^b^	25	31.3^aB^	1.6 ± 0.8^a^	7	8.8^aC^	0.4 ± 0.8^a^
**Flemish**	111	62.4^aA^	7.3 ± 2.9^a^	47	26.4^aB^	3.1 ± 0.8^a^	20	1.1^aC^	1.3 ± 0.8^a^

a,b: numbers with distinct superscripts in each column differ (P&lt;0.05); A,B,C: numbers with distinct superscripts in each row differ (P&lt;0.05). ^†^Total of 16 SOV procedures in donors from each breed, with eight females per breed for two SOV procedures per female. ‡UFO: unfertilized oocytes.

**Table 6:** Total number, mean number (LSM ± SEM) and distribution (%) of viable embryos (Grades 1, 2, and 3) obtained from eight Flemish and eight Holstein donor females following superovulation (SOV) procedures†.

**Donors**	**Grade 1**			**Grade 2**			**Grade 3**		
	**n**	**%**	**Mean**	**n**	**%**	**Mean**	**n**	**%**	**Mean**
**Holstein**	22	27.5^aA^	1.8 ± 1.4^b^	18	22.5^aA^	1.3 ± 0.7^a^	8	10.0^aB^	0.6 ± 0.9^a^
**Flemish**	48	27.0^aA^	3.4 ± 1.4^a^	34	19.1^aAB^	2.3 ± 0.7^a^	29	16.3^aB^	1.8 ± 0.8^a^

a,b: numbers with distinct superscripts in each column differ (P&lt;0.05); A,B: numbers with distinct superscripts in each row differ (P&lt;0.05). ^†^Total of 16 SOV procedures in donors from each breed, with eight females per breed for two SOV procedures per female.

No differences were observed between in the proportion of grades 1, 2, and 3 embryos collected from females from both breeds (Table 6). However, more grade 1 embryos per donor were collected from Flemish donor females than Holstein females, with no differences for grades 2 and 3 embryos per donor. A smaller proportion of grade 3 embryos than grades 1 and 2 embryos were recovered in Holstein females, and smaller than grade 1 embryos in Flemish donor females.

For comparison purposes, Table 7 summarizes the main results obtained after ten consecutive weekly OPU-IVF sessions and two SOV procedures in eight Holstein and eight Flemish donor females. Data for ovarian structures (follicles, CLs), recovered structures (total COCs, total ova) and Day-7 viable embryos (IVP, IVD), within each embryo production system (OPU-IVF, SOV), have already been compared and presented above. However, the end result, as the mean number of viable embryos obtained per session and per donor female can be compared in terms of efficiency. Thus, embryo yield by SOV procedures per female for Flemish donors was significantly higher than by SOV from Holstein females, and by OPU-IVF procedures in all females, irrespective of the breed base. In turn, OPU-IVF was similar in yielding Day-7 viable embryos in females from both breeds, and to SOV procedures for Holstein females, under the conditions of this study.

**Table 7:** Comparative efficiency between eight Flemish and eight Hosltein donor females submitted to 10 OPU-IVF session or two SOV consecutive procedures for the *in vitro* or *in vivo* embryo production.

**Donors**	**Embryo origin**	**Ovarian structures** **‡**		**Recovered structures** ^ɛ^		**Day-7 viable embryos** ^¥^	
		**n**	**Mean** **†**	**n**	**Mean^†^**	**n**	**Mean^†^**
**Holstein**	OPU-IVF	860	11.4	609	8.2	34	2.1^b^
	SOV	168	7.0	80	5.9	48	3.7^b^
**Flemish**	OPU-IVF	961	12.3	718	9.2	63	3.9^b^
	SOV	209	7.5	178	12.0	111	7.5^a^

a,b: numbers with distinct superscripts in each column differ (P&lt;0.05). ^†^Per session per donor. Total of 80 OPU procedures in females from each breed or 16 SOV procedures in females from each breed, with eight females per breed for 10 consecutive weekly OPU procedures or two SOV procedures per female. ^‡^Total number and mean number per session per donor of aspirated follicles by OPU procedures or of CLs estimated prior to embryo collection after SOV procedures. ^ɛ^Total number and mean number per session per donor of retrieved *cumulus*-oocyte complexes (COCs) after OPU procedures or of recovered structures following uterine flushing after SOV procedures. ^¥^Embryo yield on Day 7 (total number and mean number per session per donor) after *in vitro* fertilization and culture (OPU-IVF procedures, blastocyst stage only) or after embryo flushing (SOV, compact morulas and blastocysts).

## Discussion

Even though several comparative studies exist explaining mechanisms involved in the differences in performance following *in vivo* or *in vitro* embryo production systems between *Bos taurus taurus* and/or *Bos taurus indicus* (Guimarães et al., 2020; Vasconcelos et al., 2020), few studies have directly focused on reporting differences between indigenous and commercial cattle breeds from the same genetic group (Lopes da Costa et al., 2001). In this study, the group of females from the Flemish breed had a superior response and overall efficiency when compared with the group of Holstein females, especially in terms of embryo yield after either OPU-IVF or SOV procedures, even though the mean number of ovarian follicles prior to OPU and the mean number of CLs after SOV were similar between females, on a per donor basis. Differences were more pronounced in terms of total number of retrieved COCs and Day-7 structures, and developing embryos under both procedures, although the proportions of viable embryos on Day 7 were also similar between females from both breeds.

Flemish donor females had higher numbers of aspirable follicles per donor than Holstein females, as well as of recovered COCs, viable COCs, Day-7 morulas/blastocysts in both procedures, and blastocyst rates after OPU-IVF. Such findings corroborate with previous studies in which the follicular population, the total retrieved COCs and the blastocyst number after OPU-IVF had positive correlations with one another, regardless of the cattle breed (Monteiro et al., 2017). Goetten et al. (2015), comparing follicular development and P4 profiles in multiparous non-lactating Flemish and Holstein cows throughout an estrous cycle during the fall season reported no differences in the follicular dynamic patterns and P4 profiles between breeds. Pontes et al. (2009) stated that the greater the number of follicular waves, the greater the probability of finding a significant number of smaller size follicles. Based on such considerations, we infer that the follicular population and the rate of recovered COCs by OPU should be similar between Flemish and Holstein females.

Even under identical environmental and nutritional conditions, our findings highlight the differences between females from both breeds in terms of the potential as donors to provide developmentally competent oocytes. In this line, differences between donor females from both breeds in this study appeared to be a reflection of poorer oocyte competence in Holstein females. It is already known that lactating Holstein females may have compromised oocyte competence and uterine environment caused by metabolic imbalances and demands due to lactation (Lafontaine et al., 2023). Holstein cattle have been selected over the past century as a high producing milking breed, which exposes females to such metabolic stress to affect fertility more than other breeds of cattle (Gross, 2023). As Flemish cattle is still a dual purpose breed, more inclined to milk production (Thaler et al., 1996; Goetten et al., 2015), the metabolic profile and demands may not be as intense to compromise oocyte competence and fertility in milking females as in Holstein milking cows. Such speculation could explain, perhaps in part, the differences in embryo development seen between breeds used in this study, as ovarian responses were similar, but differences in COC quality and in embryo development, kinetics and quality appeared to reflect lower oocyte competence in Holstein females. However, such concepts need to be further evaluated.

The differences in aspirable follicles and number of retrieved COCs in this study may be related to greater ovarian reserve of small follicles in Flemish cows. Previously, it has been reported the existence of significant differences in preantral follicular (PAF) populations between Angus (285,155 PAFs; Silva-Santos et al., 2011) and Holstein (102,000 PAFs; Tanaka et al., 2001) females, which are breeds of similar genetic origin, although selected for distinctive production purposes. Moreover, the antral follicle count (AFC) has been shown to have a high correlation with greater circulating levels of Anti-Müllerian hormone (AMH) and IGF-1 in *Bos taurus taurus* and *Bos taurus indicus* females (Baldrighi et al., 2014; Guerreiro et al., 2014; Batista et al., 2016). In this context, we can only speculate that the higher number of oocytes recovered from Flemish than Holstein females may have been associated with IGF-1 and AMH concentration levels. As IGF-1 and AMH levels were not measured, such hypothesis still needs to be verified in the future.

Several studies have shown that COC quality is one of the key factors affecting early embryo development (Seneda et al., 2001; Pontes et al., 2010). Factors such as the operator ability and the vacuum pressure set during OPU sessions in cattle may have a direct effect on the COC quality due the pressure effect on the integrity of the *cumulus* cell layer (Manik et al., 2003). In the present study, proportions of grade I COCs were low and similar between females (7.5% for Flemish, and 7.9% for Holstein donors), corroborating with studies by Manik et al. (2003) in Karan Fries cattle, but being substantially lower than the report by Feres et al. (2018) in Gir cattle. In our study, the same experienced operator performed all OPU sessions, with all settings following the literature (Seneda et al., 2003). Still, mechanical effect on the *cumulus* cell layers caused by pressure changes along the aspiration system, needle diameter, and vacuum device placement during the OPU procedure have been previously proposed (Manik et al., 2003; Lopes et al., 2006).

Slaughterhouse ovaries contain a highly heterogeneous population of oocytes that can be recovered, regardless of the follicular dynamics, generally obtained in variable numbers and from cattle with diverse and unknown reproductive backgrounds (Karadjole et al., 2010). Consequently, COC selection is usually strict when compared with retrieved COCs by OPU procedures, which can have large variability in quality and viability (De Roover et al., 2008). In fact, it has been shown that COC recovery from slaughterhouse ovaries under conventional manual aspiration provides better morphology and quality than OPU-derived COCs (Lopes et al., 2006). Results from this study corroborate with such observations, as slaughterhouse-derived COCs were selected for better grades (I and II) prior to IVP procedures, resulting in higher cleavage and blastocyst rates (Control IVP group) than COCs derived by OPU, for which, all morphologically viable COCs (I to III) were used for IVP procedures.

The mean recovery rates of viable COCs per OPU session were similar between females (Flemish donors, 8.0 ± 0.7; Holstein donors, 7.3 ± 0.7) and to what has been already reported for other *Bos taurus taurus* breeds (Steinhauser et al., 2018; Naranjo-Chacón et al., 2019), but were lower than *Bos taurus indicus* and *Bos taurus taurus-Bos taurus indicus* crosses (12.1 ± 3.9 in Gir; 16.8 ± 5.0 in ¼ Holstein-¾ Gir; 24.3 ± 4.7 in ½ Holstein-½ Gir; Pontes et al., 2010). Interestingly, blastocyst yield in Holstein females in this study (7.2%) were similar to what was obtained in milking Holstein-Gir crosses, as mentioned above (3.2%; 3.9%; 5.5%, respectively). Such findings are in agreement with other studies under field conditions for *Bos taurus indicus* and *Bos taurus taurus*, suggesting that *in vitro* embryo production systems yield similar and variable results, regardless of the breed of the oocyte donor (Guimarães et al., 2020).

In the present study, the cleavage rates derived after OPU-IVF in Flemish cows (49.6%) were in agreement with results (48.7%) by Rizos et al. (2005), but lower than reported (79.2%) by Tamassia et al. (2003), both in Holstein. A low cleavage rate (32.8%) was observed in zygotes derived from Holstein cows. Likely, the lower COC quality obtained after OPU in Holstein females reflected a lower oocyte competence and, subsequently, low cleavage rate and blastocyst rates (7.2%, in Holstein vs 11.8% in Flemish), and slower embryo kinetics. However, blastocyst rates in Holstein cows were similar to results reported by Rizos et al. (2005) and Aller et al. (2010), i.e., 8.1% and 5.5%, respectively, but lower than what was reported (28.8%) by Tamassia et al. (2003), all in Holstein females. Even though our results are in agreement with some studies, having scientific and preservation value, cleavage and blastocyst rates in this study were below what is commercially acceptable (Oliveira et al., 2019). Nevertheless, the divergence in results observed in other studies (Pontes et al., 2010; Guerreiro et al., 2014) may be associated with metabolic profiles, high variation in COC quality and other potential factors, rather than with a breed factor.

It is well accepted that bovine IVD embryos are of better quality and have higher *in vivo* viability than IVP embryos (Hansen, 2020). Overall, several studies have reported an average of 4.5 to 6.9 transferable IVD embryos in *Bos taurus taurus* and *Bos taurus indicus* breeds (Steinhauser et al., 2018; Naranjo-Chacón et al., 2019). In the present study, the mean number of viable IVD embryos per Flemish donor fell well within data from the literature (7.3 ± 2.9). Conversely, the mean number of viable IVD embryos from Holstein donors was lower (3.3 ± 2.9) than the Flemish female counterparts and the reports from the literature, although close to values previously reported for other cattle breeds (Naranjo Chacón et al., 2020; Facioli et al., 2020). Interestingly, the mean number of CLs per donor (14.0 ± 1.8 vs. 11.3 ± 1.8) and the proportion of viable embryos over the total number of recovered structures (62.4% vs. 60.0%) were similar between females from both breeds, demonstrating that the response to the hormonal treatment and embryo development were similar between females, irrespective of the breed base. Nevertheless, the proportion of recovered structures over the CL number after flushing and the mean number of embryos per donor were lower in Holstein females than Flemish female counterparts (47.6 *vs*. 85.2%, and 5.4 ± 2.4 *vs*. 11.8 ± 2.4, respectively). Reasons for such findings still need to better elucidated.

Female response to SOV procedures is individual and quite unpredictable (Mikkola and Taponen, 2017). Previously, González et al. (1994) showed a reduced rate of fertilized COCs and transferable embryos when crude pituitary extracts containing both FSH and LH were used for SOV. However, other reports showed no detrimental effects on COCs and embryo quality using the same hormone composition (Mikkola and Taponen, 2017). Recently, Bó and Mapletoft (2020) proposed that the poor response to exogenous pituitary gonadotropins such as purified porcine pituitary-derived follicle stimulating hormone (NIH-FSH-P1/NIH-LH-S19), as used in this study, may be due to an excess of LH. Such possibility is likely associated with an individual response, health and metabolic status, and also a breed variation, as responses may be different according to the parameter, as seen in this study. The high individual and breed-specific variabilities may be due to different responses to the ovarian stimulation at the cellular and molecular levels, perhaps more evident in lactating Holstein females. Interestingly, Torres-Simental et al. (2021) showed that Simmental cows with higher AMH levels (&gt;400 pg/mL) resulted in higher AFC mean values, corpora lutea, retrieved structures and transferable embryos after superovulation. In fact, plasma AMH concentrations seem to be associated with superovulatory response (Souza et al., 2015; Aziz et al., 2017). However, AMH and IGF1 profiles between the females and/or the breeds used in this study still need to be determined.

The proportion of unfertilized oocyte (UFO) per total recovered structures was similar (50%) for females from both breeds, being similar to other reports (Sartori et al., 2002). In agreement with our findings, lower fertilization rates in superovulated cows have been reported when the AI was performed in the first 12 h after the onset of estrus than when cows were inseminated 24 hour afterwards (Dalton et al., 2000). According to Yadav et al. (1986), the estimated time for the onset of ovulations under standard superovulation protocol is 24 h after the visual observation of estrus, with all ovulations occurring during the first 11 hours after the first ovulation (35 hours after the onset of estrus). In contrast, in the present study, the AI times of the superovulated donors were 12 h and 24 h after the onset of estrus, based on visual observation twice or three times daily. Thus, the expected UFO recovery rate should have been overcome. Perhaps, UFO may be related to COCs that ovulated early and underwent aging prior to the completion of sperm transport, capacitation and fertilization.

Regardless the differences observed in donor females, it should be considered that the developmental competence of COCs and embryo viability are associated with the genetic features of each donor female, if one is to use such procedures in germoplasm conservation programs (Pontes et al., 2009; Vasconcelos et al., 2020), as the one applied to Flemish cattle in Southern Brazil. In turn, the efficiency and success of embryo production by IVF and/or SOV will depend on the costs to produce a live calf (Facioli et al., 2020). The main limitation of the current study is that the findings were generated from a small sample of animals, which is in turn of great limitation for inferences about a breed effect. Even though repetitive consecutive OPU-IVF and SOV procedures were performed in each donor female, individual variation has a larger impact on results for a smaller sample size. Indeed, sample size limitations are common in such conditions, and in studies with native cattle breeds, as shown by others (Mastromonaco and Gonzalez-Grajales, 2020; Sunds et al., 2021). The remaining Brazilian Flemish herd is already threatened by extinction and inbreeding, and targeting available specimens to conservation programs, such as the one in France (Lauvie et al., 2008; Marian et al., 2023) and under this study, justifies efforts to better understand responses to reproductive technologies.

## Conclusion

In summary, bovine donor females responded differently to the embryo production system, with females from the Flemish group producing more viable embryos by OPU-IVF and SOV procedures than females from the Holstein group. In addition, more aspirated follicles, and more total and viable COCs were recovered per OPU session in donor females from the Flemish group than from the Holstein group. Overall, SOV was more efficient than OPU/IVF in generating viable embryos per donor per session, especially in Flemish donors.

## References

[B001] Alfonzo EPM, McManus CM, Campos GS, Portes JV, Padilha AH, Peripolli V, Braccini J (2021). Spatial distribution of Brazilian bovine taurine breeds associated with climatic, physical and socioeconomic variables. Arq Bras Med Vet Zootec.

[B002] Aller JF, Mucci NC, Kaiser GG, Ríos G, Callejas SS, Alberio RH (2010). Transvaginal follicular aspiration and embryo development in superstimulated early postpartum beef cows and subsequent fertility after artificial insemination. Anim Reprod Sci.

[B003] Aziz RLA, Khalil A, Abdel-Wahab A, Hassan NY, Abdel-Hamied E, Kasimanickam RK (2017). Relationship among circulating anti-Müllerian hormone, insulin like growth factor 1, cadmium and superovulatory response in dairy cows. Theriogenology.

[B004] Baldrighi JM, Sá MF, Batista EOS, Lopes RNVR, Visintin JA, Baruselli PS, Assumpção MEOA (2014). Anti-mullerian hormone concentration and antral ovarian follicle population in Murrah heifers compared to Holstein and Gyr kept under the same management. Reprod Domest Anim.

[B005] Batista EO, Guerreiro BM, Freitas BG, Silva JC, Vieira LM, Ferreira RM, Rezende RG, Basso AC, Lopes RN, Rennó FP, Souza AH, Baruselli PS (2016). Plasma anti-Müllerian hormone as a predictive endocrine marker to select Bos taurus (Holstein) and Bos indicus (Nelore) calves for in vitro embryo production. Domest Anim Endocrinol.

[B006] Bó GA, Baruselli PS, Chesta PM, Martins CM (2006). The timing of ovulation and insemination schedules in superstimulated cattle. Theriogenology.

[B007] Bó GA, Mapletoft RJ (2020). Superstimulation of ovarian follicles in cattle: gonadotropin treatment protocols and FSH profiles. Theriogenology.

[B008] Clasen JB, Kargo M, Fikse WF, Strandberg E, Wallenbeck A, Østergaard S, Rydhmer L (2021). Conservation of a native dairy cattle breed through terminal crossbreeding with commercial dairy breeds. Acta Agric Scand A Anim Sci.

[B009] Cruz FB, Oliveira I, Vieira AD, Gerger RPC, Ribeiro ES, Bertolini M, Mezzalira A (2008). Recoleta uterina como estratégia para aumentar a taxa de embriões em fêmeas bovinas de corte e leite. Acta Sci Vet.

[B010] Dalton JC, Nadir S, Bame JH, Noftsinger M, Saacke RG (2000). The effect of time of artificial insemination on fertilization status and embryo quality in superovulated cows. J Anim Sci.

[B011] De Roover R, Feugang JMN, Bols PEJ, Genicot G, Hanzen C (2008). Effects of ovum pick-up frequency and fsh stimulation: a retrospective study on seven years of beef cattle *in vitro* embryo production. Reprod Domest Anim.

[B012] Facioli FL, De Marchi F, Marques MG, Michelon PRP, Zanella EL, Caires KC, Reeves JJ, Zanella R (2020). The outcome and economic viability of embryo production using IVF and SOV techniques in the wagyu breed of cattle. Vet Sci.

[B013] Feres LF, Siqueira LGB, Palhao MP, Santos LL, Brandao FZ, Viana JHM (2018). Likelihood of pregnancy after the transfer of embryos derived from follicle aspiration and *in vitro* embryo production sessions with different relative efficiencies. Anim Reprod Sci.

[B014] Gicquel E, Boettcher P, Besbes B, Furre S, Fernández J, Danchin-Burge C, Berger B, Baumung R, Feijóo J, Leroy G (2020). Impact of conservation measures on demography and genetic variability of livestock breeds. Animal.

[B015] Godde CM, Mason-D’Croz D, Mayberry DE, Thornton PK, Herrero M (2021). Impacts of climate change on the livestock food supply chain; a review of the evidence. Glob Food Sec.

[B016] Goetten ALF, Mezzalira A, Zago FC, Portela VM (2015). Characterization of the follicular dynamic patterns in a red flemish herd in Southern Brazil. Acta Sci Vet.

[B017] González A, Wang H, Carruthers T, Murphy B, Mapletoft R (1994). Increased ovulation rates in PMSG-stimulated beef heifers treated with a monoclonal PMSG antibody. Theriogenology.

[B018] Gross JJ (2023). Dairy cow physiology and production limits. Anim Front.

[B019] Guerreiro BM, Batista EO, Vieira LM, Sá MF, Rodrigues CA, Castro A, Silveira CR, Bayeux BM, Dias EA, Monteiro FM, Accorsi M, Lopes RN, Baruselli PS (2014). Plasma anti-mullerian hormone: an endocrine marker for in vitro embryo production from Bos taurus and Bos indicus donors. Domest Anim Endocrinol.

[B020] Guimarães ASB, Rocha LF, Jesus RDL, Vasconcelos GL, Anghinoni G, Santana ALA, Barbosa LP (2020). In vitro performance of Zebu (*Bos indicus*) and Taurus (*Bos taurus*) donor cow embryos. Rev Bras Saúde Prod Anim.

[B021] Hansen PJ (2020). The incompletely fulfilled promise of embryo transfer in cattle-why aren’t pregnancy rates greater and what can we do about it?. J Anim Sci.

[B022] Holm P, Booth PJ, Schmidt MH, Greve T, Callesen H (1999). High bovine blastocyst development in a static *in vitro* production system using SOFaa medium supplemented with sodium citrate and myo-inositol with or without serum-proteins. Theriogenology.

[B023] Karadjole M, Getz I, Samardžija M, Maćešić N, Matkovi M, Makek Z, Karadjole T, Bačić G, Dobrani T, Poletto M (2010). The developmental competence of bovine immature oocytes and he developmental competence of bovine immature oocytes and quality of embryos derived from slaughterhouse ovaries or live uality of embryos derived from slaughterhouse ovaries or live donors by ovum pick up onors by ovum pick up. Vet Arh.

[B024] Lafontaine S, Labrecque R, Blondin P, Cue RI, Sirard MA (2023). Comparison of cattle derived from in vitro fertilization, multiple ovulation embryo transfer, and artificial insemination for milk production and fertility traits. J Dairy Sci.

[B025] Lauvie A, Danchin-Burge C, Audiot A, Brives H, Casabianca F, Verrier E (2008). A controversy about crossbreeding in a conservation programme: the case study of the Flemish Red cattle breed. Livest Sci.

[B026] Liu X, Li Z, Yan Y, Li Y, Wu H, Pei J, Yan P, Yang R, Guo X, Lan X (2021). Selection and introgression facilitated the adaptation of Chinese native endangered cattle in extreme environments. Evol Appl.

[B027] Lopes AS, Martinussen T, Greve T, Callesen H (2006). Effect of days post-partum, breed and ovum pick-up scheme on bovine oocyte recovery and embryo development. Reprod Domest Anim.

[B028] Lopes da Costa  L, Chagas e Silva J, Silva Robalo J (2001). Superovulatory response, embryo quality and fertility after treatment with different gonadotrophins in native cattle. Theriogenology.

[B029] Machado GM, Carvalho JO, Siqueira E, Caixeta ES, Franco MM, Rumpf R, Dode MAN (2009). Effect of Percoll volume, duration and force of centrifugation, on *in vitro* production and sex ratio of bovine embryos. Theriogenology.

[B030] Manik RS, Singla SK, Palta P (2003). Collection of oocytes through transvaginal ultrasound-guided aspiration of follicles in an Indian breed of cattle. Anim Reprod Sci.

[B031] Marian L, Withoeft JA, Costa LDS, Ribeiro LR, Melo IC, Alves RS, Baumbach LF, Pinto MGL, Snak A, Miletti LC, Ferraz SM, Sfaciotte RAP, Canal CW, Casagrande RA (2023). Causes of fetal death in the Flemish cattle herd in Brazil. Vet World.

[B032] Mastromonaco GF, Gonzalez-Grajales AL (2020). Reproduction in female wild cattle: influence of seasonality on ARTs. Theriogenology.

[B033] Mikkola M, Taponen J (2017). Embryo yield in dairy cattle after superovulation with Folltropin or Pluset. Theriogenology.

[B034] Monteiro FM, Batista E, Vieira LM, Bayeux BM, Accorsi M, Campanholi SP, Dias E, Souza AH, Baruselli PS (2017). Beef donor cows with high number of retrieved COC produce more *in vitro* embryos compared with cows with low number of COC after repeated ovum pick-up sessions. Theriogenology.

[B035] Naranjo Chacón F, Montiel Palacios F, Canseco Sedano R, Ahuja-Aguirre C (2020). Embryo production after superovulation of bovine donors with a reduced number of FSH applications and an increased eCG dose. Theriogenology.

[B036] Naranjo-Chacón F, Montiel-Palacios F, Canseco-Sedano R, Ahuja-Aguirre C (2019). Embryo production in middle-aged and mature *Bos taurus* × *Bos indicus* cows induced to multiple ovulation in a tropical environment. Trop Anim Health Prod.

[B037] Oliveira ATD, Lopes RFF, Rodrigues JL (2006). Gene expression and developmental competence of bovine embryos produced *in vitro* with different serum concentrations. Reprod Domest Anim.

[B038] Oliveira SC, Serapião RV, Camargo AJR, Freitas C, Iguma LT, Carvalho BC, Camargo LSA, Oliveira LZ, Verneque RS (2019). Oocyte origin affects the *in vitro* embryo production and development of Holstein (*Bos taurus taurus*) - Gyr (*Bos taurus indicus*) reciprocal cross embryos. Anim Reprod Sci.

[B039] Parra GAC, Taramuel LGC, Bustos JJG (2020). Evaluation of the productive characteristics of the Caqueteño Creole cattle breed. Trop Anim Health Prod.

[B040] Parrish JJ, Susko-Parrish J, Winer MA, First NL (1988). Capacitation of bovine sperm by heparin. Biol Reprod.

[B041] Pieterse MC, Kappen KA, Kruip TAM, Taverne MAM (1988). Aspiration of bovine oocytes during transvaginal ultrasound scanning of the ovaries. Theriogenology.

[B042] Pontes JH, Nonato-Junior I, Sanches BV, Ereno-Junior JC, Uvo S, Barreiros TR, Oliveira JA, Hasler JF, Seneda MM (2009). Comparison of embryo yield and pregnancy rate between *in vivo* and *in vitro* methods in the same Nelore (*Bos indicus*) donor cows. Theriogenology.

[B043] Pontes JH, Silva KC, Basso AC, Rigo AG, Ferreira CR, Santos GM, Sanches BV, Porcionato JP, Vieira PH, Faifer FS, Sterza FA, Schenk JL, Seneda MM (2010). Large-scale *in vitro* embryo production and pregnancy rates from *Bos taurus, Bos indicus*, and indicus-taurus dairy cows using sexed sperm. Theriogenology.

[B044] Ribeiro ES, Gerger RPC, Ohlweiler LU, Ortigari I, Mezzalira JC, Forell F, Bertolini LR, Rodrigues JL, Ambrósio CE, Miglino MA, Mezzalira A, Bertolini M (2009). Developmental potential of bovine hand-made clone embryos reconstructed by aggregation or fusion with distinct cytoplasmic volumes. Cloning Stem Cells.

[B045] Rizos D, Burke L, Duffy P, Wade M, Mee JF, O’Farrell KJ, MacSiurtain M, Boland MP, Lonergan P (2005). Comparisons between nulliparous heifers and cows as oocyte donors for embryo production *in vitro.*. Theriogenology.

[B046] Sartori R, Sartor-Bergfelt R, Mertens SA, Guenther JN, Parrish JJ, Wiltbank MC (2002). Fertilization and early embryonic development in heifers and lactating cows in summer and lactating and dry cows in winter. J Dairy Sci.

[B047] Seneda MM, Esper CR, Garcia JM, Andrade ER, Binelli M, Oliveira JA, Nascimento AB (2003). Efficacy of linear and convex transducers for ultrasound-guided transvaginal follicle aspiration. Theriogenology.

[B048] Seneda MM, Esper CR, Garcia JM, Oliveira JA, Vantini R (2001). Relationship between follicle size and ultrasound-guided transvaginal oocyte recovery. Anim Reprod Sci.

[B049] Silva-Santos KC, Santos GM, Siloto LS, Hertel MF, Andrade ER, Rubin MI, Sturion L, Melo-Sterza FA, Seneda MM (2011). Estimate of the population of preantral follicles in the ovaries of *Bos taurus indicus* and *Bos taurus taurus* cattle. Theriogenology.

[B050] Souza AH, Carvalho PD, Rozner AE, Vieira LM, Hackbart KS, Bender RW, Dresch AR, Verstegen JP, Shaver RD, Wiltbank MC (2015). Relationship between circulating anti-Müllerian hormone (AMH) and superovulatory response of high-producing dairy cows. J Dairy Sci.

[B051] Steinhauser CB, Looney CR, Hasler JF, Renaud P (2018). Retrospective analysis of superstimulation with Folltropin^®^-V in Wagyu versus other beef breeds. Anim Reprod.

[B052] Stojkovic M, Machado SA, Stojkovic P, Zakhartchenko V, Hutzler P, Goncalves PB, Wolf E (2001). Mitochondrial distribution and adenosine triphosphate content of bovine oocytes before and after in vitro maturation: correlation with morphological criteria and developmental capacity after in vitro fertilization and culture. Biol Reprod.

[B053] Stringfellow DA, Givens MD (2009). Manual of the International Embryo Transfer Society: a procedural guide and general information for the use of embryo transfer technology emphasizing sanitary procedures.

[B054] Sunds AV, Bunyatratchata A, Robinson R, Glantz M, Paulsson M, Leskauskaite D, Pihlanto A, Inglingstad R, Devold TG, Vegarud GE, Birgisdottir BE, Gudjonsdottir M, Barile D, Larsen LB, Poulsen NA (2021). Comparison of bovine milk oligosaccharides in native North European cattle breeds. Int Dairy J.

[B055] Taberlet P, Valentini A, Rezaei HR, Naderi S, Pompanon F, Negrini R, Ajmone-Marsan P (2008). Are cattle, sheep, and goats endangered species?. Mol Ecol.

[B056] Tamassia M, Heyman Y, Lavergne Y, Richard C, Gelin V, Renard JP, Chastant-Maillard S (2003). Evidence of oocyte donor cow effect over oocyte production and embryo development *in vitro.*. Reproduction.

[B057] Tanaka Y, Nakada K, Moriyoshi M, Sawamukai Y (2001). Appearance and number of follicles and change in the concentration of serum FSH in female bovine fetuses. Reproduction.

[B058] Thaler A, Mühibauer MD, Ramos JC, Zardo WF (1996). Fatores que afetam a produção de leite e o período de lactação em um rebanho das raças Flamenga e Holandesa no Planalto Catarinense. Cienc Rural.

[B059] Torres-Simental JF, Peña-Calderón C, Avendaño-Reyes L, Correa-Calderón A, Macías-Cruz U, Rodríguez-Borbón A, Leyva-Corona JC, Rivera-Acuña F, Thomas MG, Luna-Nevárez P (2021). Predictive markers for superovulation response and embryo production in beef cattle managed in northwest Mexico are influenced by climate. Livest Sci.

[B060] Vajta G, Holm P, Greve T, Callesen H (1997). The submarine incubation system, a new tool for *in vitro* embryo culture: a technique report. Theriogenology.

[B061] Vasconcelos GL, Cunha EV, Maculan R, Sánchez Viafara JA, Silva AWB, Batista ALS, Silva JRV, Souza JC (2020). Effects of vulvar width and antral follicle count on oocyte quality, *in vitro* embryo production and pregnancy rate in Bos taurus taurus and Bos taurus indicus cows. Anim Reprod Sci.

[B062] Yadav MC, Walton JS, Leslie KE (1986). Timing of the onset and duration of ovulation in superovulated beef heifers. Theriogenology.

[B063] Zander KK, Signorello G, Salvo M, Gandini G, Drucker AG (2013). Assessing the total economic value of threatened livestock breeds in Italy: implications for conservation policy. Ecol Econ.

